# Diagnostic performance of ACR-TIRADS combined with superb microvascular imaging for differential diagnosis of mummified thyroid nodules and papillary thyroid carcinomas

**DOI:** 10.1530/EC-23-0388

**Published:** 2024-02-07

**Authors:** Jiali Tian, Jinlei Liang, Yuhong Lin, Liping Wang, Xiaobo Chen

**Affiliations:** 1 Ultrasound Department, The Fifth Affiliated Hospital of Sun Yat-Sen University, Zhuhai, China; 2 Ultrasound Department, Zhuhai People's Hospital, Zhuhai, China; 3 Ultrasound Department, Zhuhai Xiangzhou District People's Hospital, Zhuhai, China

**Keywords:** mummified thyroid nodule, papillary thyroid carcinoma, thyroid imaging reporting and data system, superb microvascular imaging, diagnostic value

## Abstract

**Objective:**

The aim was to investigate the ability of superb microvascular imaging (SMI) to improve the differential diagnosis of mummified thyroid nodules (MTNs) and papillary thyroid carcinomas (PTCs) using the 2017 American College of Radiology Thyroid Imaging Reporting and Data System (ACR-TIRADS).

**Materials and methods:**

We enrolled 110 cases of MTNs and 110 cases of PTCs confirmed by fine needle aspiration (FNA) or surgery. Conventional ultrasound (US) and the quantity of microvessels detected by SMI were analyzed for all nodules. Thyroid nodules were initially categorized by ACR-TIRADS based on US imaging features and then reclassified based on ACR-TIRADS combined with SMI blood-flow grade (SMI-TIRADS). We compared the diagnostic performances of ACR-TIRADS and SMI-TIRADS by receiver operating characteristic curve, sensitivity, specificity, accuracy, positive predictive value (PPV), and negative predictive value (NPV).

**Results:**

US-detected margin, shape, and echogenic foci differed between MTNs and PTCs (*P <* 0.05). The SMI blood-flow grade was significantly greater in PTCs compared with MTNs (*Χ*^2^ = 158.78, *P* < 0.05). There was no significant difference in ACR-TIRADS indicators between MTNs and PTCs (*Χ*^2^ = 1.585, *P* = 0.453); however, reclassification by SMI-TIRADS showed significant differences between the groups (*Χ*^2^ = 129.521, *P* < 0.001). The area under the curve was significantly lower for ACR-TIRADS compared with SMI-TIRADS (0.517 vs 0.887, *P* < 0.05). SMI-TIRADS had significantly higher diagnostic value for distinguishing MTNs and PTCs than ACR-TIRADS (sensitivity: 91.82% vs 74.55%, *P <* 0.05; specificity: 84.55% vs 21.82%, *P* < 0.05; accuracy: 88.18% vs 48.18%, *P* < 0.05; PPV: 85.59% vs 48.81%, *P* < 0.05; and NPV: 91.18% vs 46.15%, *P* < 0.05).

**Conclusion:**

The detection of microvascular flow and large vessels in thyroid nodules by SMI resulted in high diagnostic specificity and sensitivity. ACR-TIRADS combined with SMI could effectively distinguish between MTNs and PTCs, to avoid unnecessary FNA or surgical excision.

## Introduction

Thyroid nodules (TNs) are common endocrine masses that can be detected by palpation in approximately 4–7% of cases; however, ultrasound (US) examination of the thyroid is ten times more sensitive than palpation and can detect 19–67% of TNs (1, 2, 3, 4, 5, 6). The American College of Radiology published its Thyroid Imaging Reporting and Data System (ACR-TIRADS) in 2017 to stratify TNs based on US features (7). Each TIRADS score indicates a different malignancy risk, and the risk stratification system thus allows the standardized evaluation of TNs (7). However, various changes may occur in benign cystic or mixed cystic and solid TNs, such as centronodular thyrocyte hypoxia, nodule hemorrhage, absorption of cystic fluid over time, and effects of thyroid nodule fine needle aspiration (FNA) or radiofrequency ablation, and the nodules may become dry, shrunken, and solid. Lacout *et al.* referred to these as mummified TNs (MTNs) (8). According to ACR-TIRADS, MTNs should have at least two suspicious US features suggestive of malignancy, including a gently irregular margin, solid composition, hypoechoic echotexture, and punctate echogenic foci suggestive of microcalcifications (9, 10, 11). In patients with no definite medical history and no previous US examination results, these nodules are often classified as TIRADS score 4 or 5, and may be mistaken for malignancies, leading MTN patients to undergo unnecessary repeated FNA procedures, or even surgery (12). It is difficult to distinguish these nodules from papillary thyroid carcinomas (PTCs) based solely on two-dimensional US features, and the diagnostic efficiency of conventional US for MTNs is limited. Previous studies showed that the vascular system in benign TNs was destroyed during mummification (11), while PTCs include abundant vasculature and branching vessels due to abnormal angiogenesis during carcinogenesis, as cancer cells invade nodule areas deficient in blood vessels (13, 14). MTNs and PTCs thus have different distributions of branching vessels and microvascularization. Previous studies found that it was difficult to differentiate the microvasculature within lesions using color Doppler flow imaging (CDFI) and power Doppler imaging (PDI) (15). Although contrast-enhanced US (CEUS) can be used to distinguish MTNs and PTCs, it is relatively invasive and the contrast agents may cause allergic reactions (16). Superb microvascular imaging (SMI) is an innovative microvascular US imaging technique that can separate low-speed flow signals from motion artifacts by multidimensional filtering (17). Machaodo *et al.* found that SMI was superior to CDFI and PDI for detecting small vessel branching (18), and SMI also improved the differentiation of benign and malignant breast lesions (19). Nevertheless, the role of SMI in distinguishing MTNs and PTCs remains unknown. This study aimed to compare the SMI blood-flow features of MTNs and PTCs and to explore the diagnostic value of ACR-TIRADS combined with SMI for the differential diagnosis of MTNs and PTCs.

## Materials and methods

### Patient selection

Patients with TNs and/or suspected thyroid cancer at the Fifth Affiliated Hospital of Sun Yat-Sen University, Zhuhai People’s Hospital, and Zhuhai Xiangzhou District People’s Hospital were recruited from June 2020 to August 2023.

### Ethics

This prospective study was approved by the Ethics Committee of the Fifth Affiliated Hospital of Sun Yat-Sen University and performed in accordance with the Declaration of Helsinki for human studies (2023(K196-1)). Informed consent was obtained from all patients before data collection.

#### Inclusion criteria

The inclusion criteria were as follows: (i) patients with suspected malignant TNs according to conventional US; (ii) patients with TNs scheduled for biopsy and/or subsequent thyroidectomy; and (iii) patients with TNs who could complete conventional US and SMI examinations.

#### Exclusion criteria

The exclusion criteria were as follows: (i) patients whose US images did not meet the quality requirements (two-dimensional or SMI images were not clear or standard); (ii) patients with thyroid parenchyma with rich blood-flow signals; (iii) patients with TNs with macrocalcifications or peripheral calcifications; (iv) pregnant or lactating patients; (v) patients who refused FNA or surgery; and (vi) patients without a conclusive diagnosis determined by cytology, histopathology, or both.

### US imaging procedures

Conventional US and SMI examinations were conducted using an Aplio 500 (Canon Medical Systems Corporation, Tokyo, Japan) equipped with a 5–14 MHz linear probe. The patients were placed in a supine position, and the neck region was fully exposed. The settings used in the study were a velocity scale of 10 cm/s for CDFI and 1.0–2.5 cm/s for the SMI modes. The main parameters were set as follows: mechanical index, 1.5; frame rate, 25–60 frames/s; and color gain, 40–80 dB. A minimum of three images of each TN were acquired in gray-scale US and SMI with monochrome mode. In gray-scale US mode, the images were acquired when the nodule demonstrated the maximum diameter, composition, echogenicity, shape, margin, and calcification. For monochrome SMI examination, the images were acquired when the nodule demonstrated abundant vascularity and stable Doppler US signals.

#### Gray-scale US assessment

All examinations were performed by a single sonographer with over 10 years of experience in thyroid US scanning. Another sonographer, who was blinded to the patients’ clinical data, analyzed the images independently to classify the TNs. The interpretations were scored based on ACR-TIRADS (20), and the TNs were evaluated according to the following five features: composition, echogenicity, shape, margin, and echogenic foci. Composition was classified as follows: cystic or almost completely cystic, TIRADS score 0; spongiform, TIRADS score 0; mixed cystic and solid, TIRADS score 1; and solid or almost completely solid, TIRADS score 2. Echogenicity was classified as follows: anechoic, TIRADS score 0; hyperechoic or isoechoic, TIRADS score 1; hypoechoic, TIRADS score 2; and very hypoechoic, TIRADS score 3. Shape was classified as: wider than tall, TIRADS score 0 and taller than wide, TIRADS score 3. Margin was classified as follows: smooth, TIRADS score 0; ill-defined, TIRADS score 0; lobulated or irregular, TIRADS score 2; and extrathyroidal extension, TIRADS score 3. Echogenic foci were classified as follows: none or large comet-tail artifacts, TIRADS score 0; macrocalcifications, TIRADS score 1; peripheral (rim) calcifications, TIRADS score 2; and punctate echogenic foci, TIRADS score 3.

#### Quantitative vascular assessment

**Figure 1:**
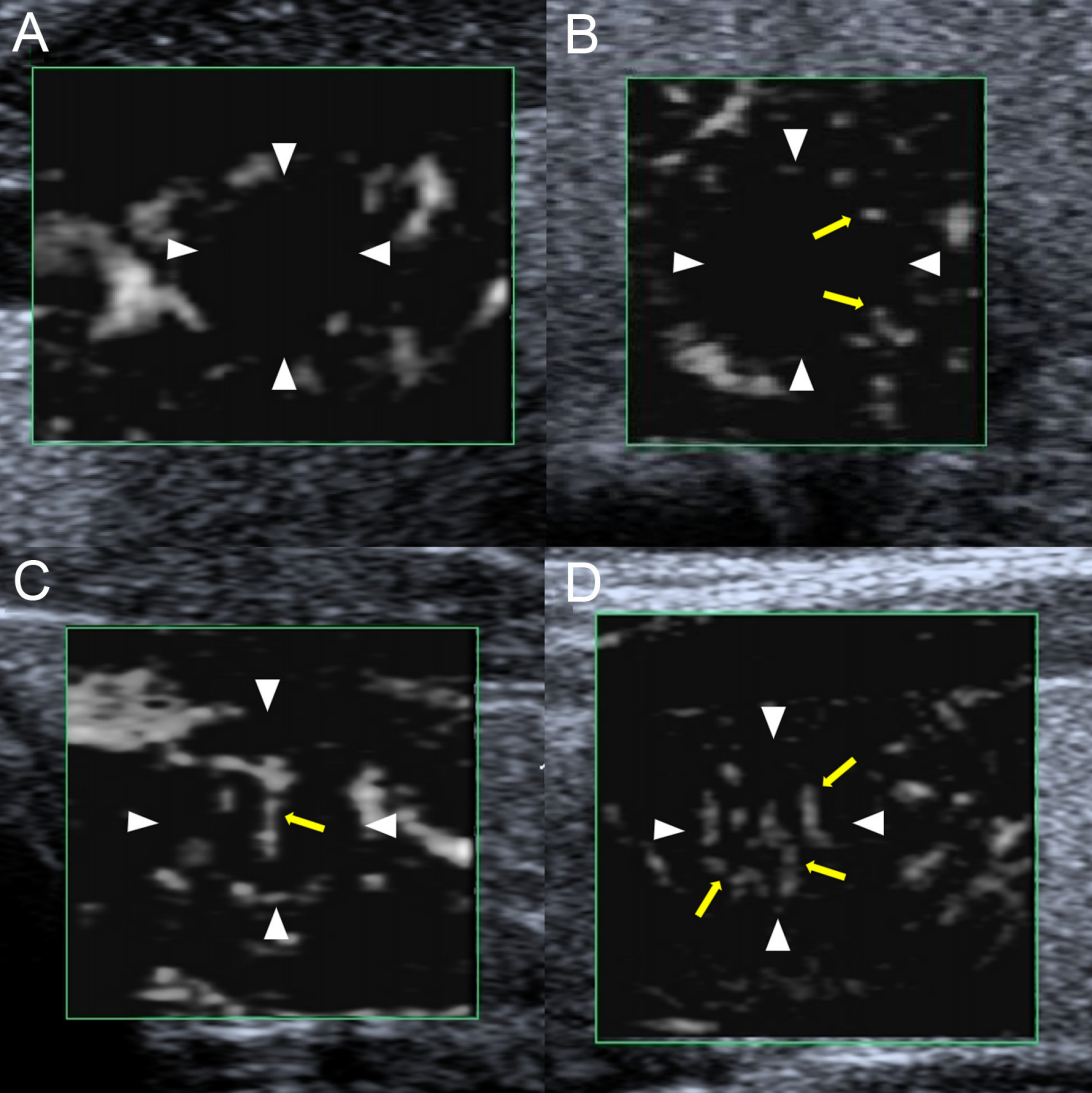
Superb microvascular imaging blood flow quantitative grading (A–D). (A) grade 0; (B) grade 1; (C) grade 2; (D) grade 3.

SMI was used to evaluate the quantity of microvessels. The subjective assessment of quantitative grading criteria was based on previous study (21) and our experience. The grading system was defined as follows: grade 0, no blood flow inside the TNs (Fig. 1A); grade 1, SMI revealed minimal blood flow (one or two pixels or rod-shaped) inside the TNs, without perforating vessels (Fig. 1B); grade 2, moderate blood flow inside the TNs, with main vessel length similar to or greater than the nodule radius in the area and/or several small vessels (Fig. 1C); and grade 3, rich blood flow inside the TNs, with four or more vessels or two longer vessels, having lengths similar to or greater than the nodule radius (Fig. 1D).

#### Final SMI-TIRADS score

If the SMI blood-flow grade of the TN was 0, one score was subtracted from the ACR-TIRADS classification. If the SMI blood-flow grade of the nodule was 2 or 3, one score was added to the ACR-TIRADS classification, but score 5 remained the same; if the SMI blood-flow grade was 1, the ACR-TIRADS classification remained the same.

#### MTN diagnostic criteria

(i) Nodules with a history of gradual shrinkage from typical benign cystic nodules or mixed cystic and solid nodules, and the nodule volume reduced by >45%; (ii) a benign diagnosis based on cytopathology or postoperative pathology (8).

#### PTC diagnostic criteria

FNA cytology or postoperative pathology.

The pathological findings of all TNs were evaluated by a pathologist with more than 10 years of experience.

### Statistical analysis

Statistical analysis was carried out using SPSS software (version 25.0) and MedCalc (version 19.8). Normally distributed data were presented as mean ± s.d. and analyzed with independent samples *t*-tests. Categorical variables were expressed as numbers and percentages and analyzed by *χ*^2^ tests. Receiver operating characteristic curves were utilized to assess the diagnostic values of SMI-TIRADS and ACR-TIRADS in distinguishing between MTNs and PTCs, and c-statistics were compared using the method of DeLong *et al.* (22). The sensitivity, specificity, accuracy, positive predictive value (PPV), and negative predictive value (NPV) were calculated at the optimal cutoff points and compared with McNemar’s test. *P* < 0.05 denoted statistical significance.

## Results

### Basic characteristics

**Figure 2:**
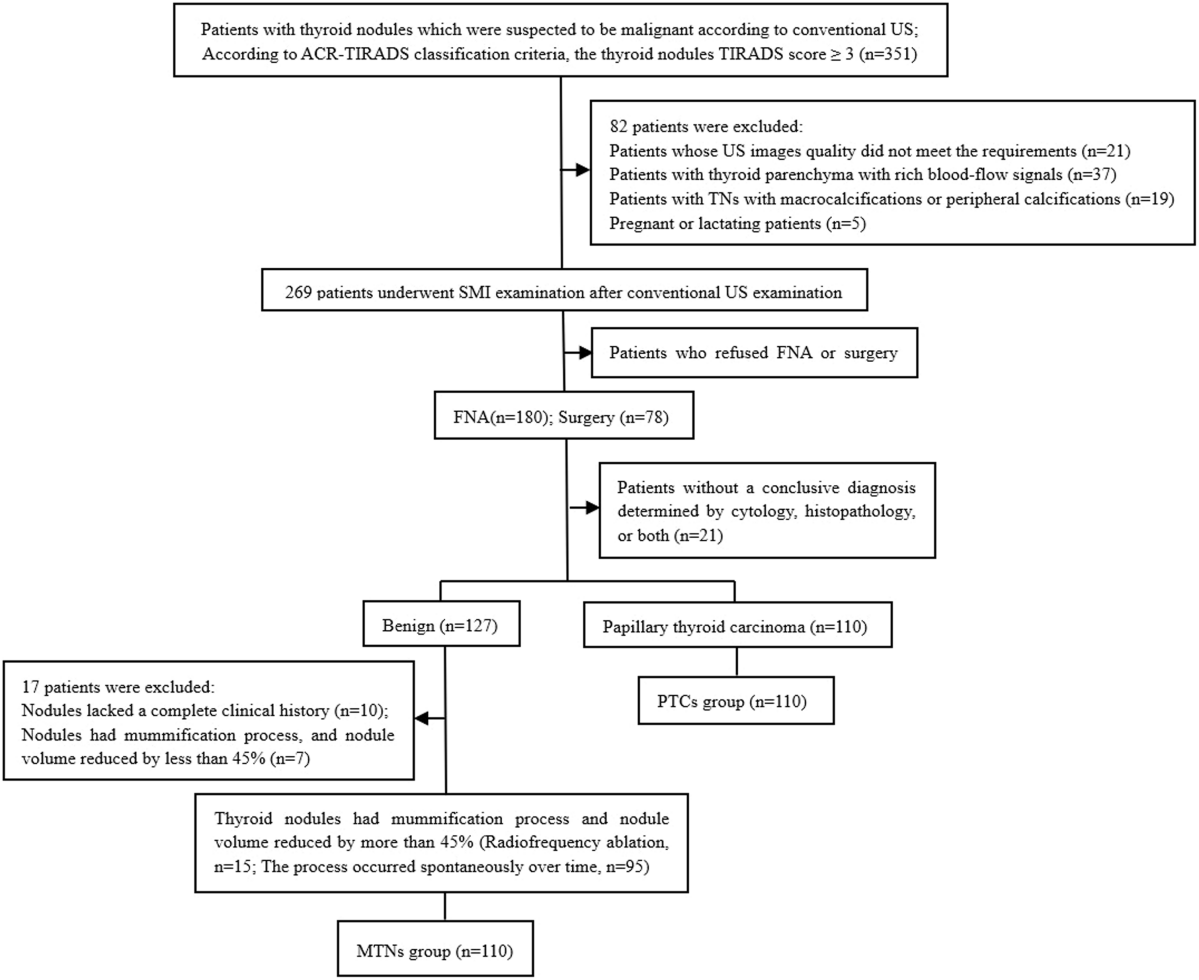
Flow diagram.

A total of 220 lesions, including 110 MTNs and 110 PTCs, were included in the study based on the pathological diagnosis or FNA cytology results. A flowchart of the study is shown in Fig. 2. The mean patient age (57 males; 163 females) was 49.01 ± 10.26 years (range 18–72). There was no significant differences in age, sex, maximum nodule diameter, composition, echogenicity, or PDI grade between the MTN and PTC groups (*P* > 0.05). Most MTNs and PTCs were hypoechoic or very hypoechoic. PTCs more commonly had irregular margins and extrathyroidal extensions, and were taller-than-wide. Most MTNs were SMI blood-flow grade 0–1 and most PTCs were grade 2–3 (*P* < 0.05). In the MTN group, there was no significant difference in blood flow grading between PDI and SMI (*χ^2^* = 0.268, *P* = 0.874). However, in the PTC group, the blood flow grade of SMI was higher than that of PDI (*χ^2^* = 136.362, *P* < 0.001) (Table 1).

**Table 1:** Basic characteristics in the MTN and PTC group.

Variable	MTN (*n* = 110)	PTC (*n* = 110)	*t/χ^2^*	*P*
Age (year)	50.21 ± 11.36	47.82 ± 8.92	1.736	0.084
Gender (*n* (%))			0.024	0.878
Male	28 (25.45)	29 (26.36)		
Female	82 (74.55)	81 (73.64)		
Maximum diameter (mm)	9.18 ± 5.79	8.93 ± 4.99	0.348	0.728
Composition (*n* (%))			0.338	0.561
Cystic/almost completely cystic	0 (0)	0 (0)		
Spongiform	0 (0)	0 (0)		
Mixed cystic and solid	1 (0.91)	2 (1.82)		
Solid/almost completely solid	109 (99.09)	108 (98.18)		
Echogenicity (*n* (%))			3.514	0.173
Anechoic	0 (0)	0 (0)		
Hyperechoic/isoechoic	0 (0)	3 (2.73)		
Hypoechoic	82 (74.55)	84 (76.36)		
Very hypoechoic	28 (25.45)	23 (20.91)		
Shape (*n* (%))			9.683	0.002
Wider than tall	62 (56.36)	39 (35.45)		
Taller than wide	48 (43.64)	71 (64.55)		
Margin (*n* (%))			36.755	<0.001
Smooth	81 (73.64)	42 (38.18)		
Ill-defined	13 (11.82)	16 (14.54)		
Lobulated/irregular	16 (14.54)	35 (31.82)		
Extrathyroidal extension	0 (0)	17 (15.46)		
Echogenic foci (*n* (%))			25.812	<0.001
None or large comet-tail artifacts	27 (24.55)	44 (40.00)		
Macrocalcifications	33 (30.00)	5 (4.55)		
Peripheral (rim) calcifications	1 (0.91)	1 (0.91)		
Punctate echogenic foci	49 (44.54)	60 (54.54)		
PDI grade (*n* (%))			6.971	0.073
Grade 0	88 (80.00)	72 (65.45)		
Grade 1	18 (16.36)	27 (24.55)		
Grade 2	4 (3.64)	10 (9.09)		
Grade 3	0 (0)	1 (0.91)		
SMI BF (*n* (%))			158.781	<0.001
Grade 0	85 (77.27)	3 (2.73)		
Grade 1	20 (18.18)	13 (11.82)		
Grade 2	5 (4.55)	48 (43.64)		
Grade 3	0 (0)	46 (41.81)		

MTNs, mummified thyroid nodules; PTCs, papillary thyroid carcinomas; PDI, power Doppler imaging; SMI BF, superb microvascular imaging blood flow.

### Comparison of MTNs and PTCs using ACR-TIRADS and SMI-TIRADS

There was no significant difference in the ACR-TIRADS scores between MTNs and PTCs (*P* > 0.05). However, MTNs and PTCs could be distinguished using the corrected SMI-TIRADS scores (*P* < 0.05) (Table 2).

**Table 2:** MTNs and PTCs classified according to the ACR-TIRADS and SMI-TIRADS values.

	Total (*n* = 220)	MTNs (*n* = 110)	PTCs (*n* = 110)	Recommended malignant risk	Calculated malignant risk	*χ^2^*	*P*
ACR-TIRADS						1.585	0.453
Score 3	1 (0.46%)	1 (0.91%)	0 (0)	<5%	0%		
Score 4	51 (23.18%)	23 (20.91%)	28 (25.45%)	5–20%	54.90%		
Score 5	168 (76.36%)	86 (78.18%)	82 (74.55%)	>20%	48.81%		
SMI-TIRADS						129.521	<0.001
Score 3	15 (6.82%)	15 (13.63%)	0 (0)	<5%	0%		
Score 4	87 (39.54%)	78 (70.91%)	9 (8.18%)	5–20%	10.34%		
Score 5	118 (53.64%)	17 (15.46%)	101 (91.82%)	>20%	85.59%		

ACR-TIRADS, American College of Radiology Thyroid Imaging Reporting and Data System; MTNs, mummified thyroid nodules; PTCs, papillary thyroid carcinomas; SMI-TIRADS, ACR-TIRADS combined with SMI blood flow grades.

### Diagnostic performances of SMI-TIRADS and ACR-TIRADS for distinguishing MTNs from PTCs

**Figure 3:**
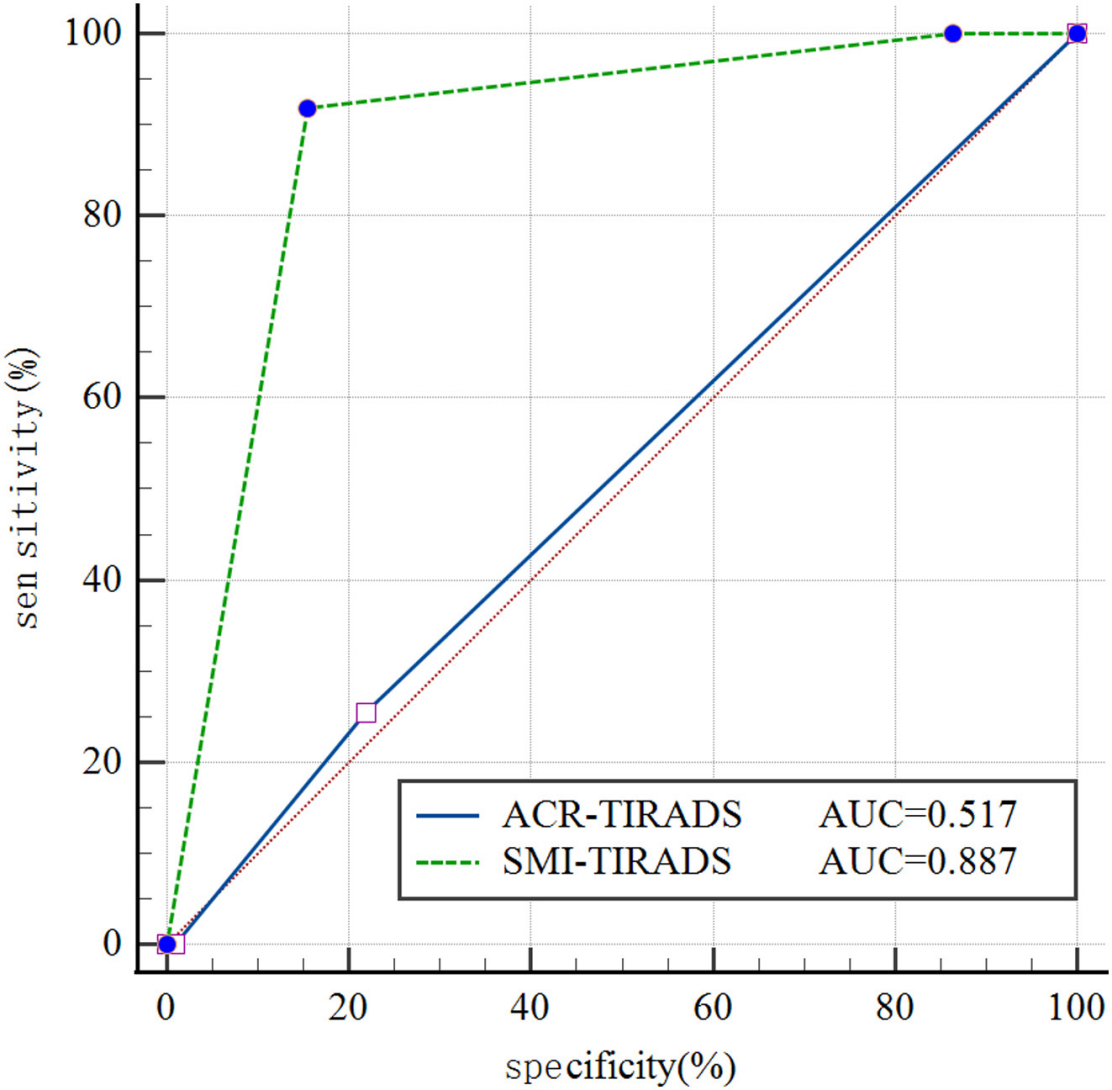
Diagnostic performance evaluation of SMI-TIRADS and ACR-TIRADS in distinguishing MTNs from PTCs ROC curve.

The areas under the curves (AUCs) for distinguishing MTNs from PTCs differed significantly between ACR-TIRADS and SMI-TIRADS (0.517 and 0.887, respectively), with the latter having significantly better diagnostic value than the former (*P* < 0.05) (Fig. 3). An SMI-TIRADS score of 5 was used as the cutoff value for distinguishing MTNs from PTCs. SMI-TIRADS scores showed a sensitivity, specificity, accuracy, PPV, and NPV of 91.82%, 84.55%, 88.18%, 85.59%, and 91.18%, respectively. These values were significantly superior to those for ACR-TIRADS (74.55%, 21.82%, 48.18%, 48.81%, and 46.15%, respectively), indicating a significantly improved diagnostic performance (Table 3).

**Table 3:** Diagnostic performance evaluation of SMI-TIRADS and ACR-TIRADS in distinguishing MTNs and PTCs.

Method	Cutoff score	Sensitivity (%)	Specificity (%)	Accuracy (%)	PPV (%)	NPV (%)	AUC (95% CI)
ACR-TIRADS	TIRADS 4	74.55%	21.82%	48.18%	48.81%	46.15%	0.517 (0.449–0.585)
SMI-TIRADS	TIRADS 5	91.82%	84.55%	88.18%	85.59%	91.18%	0.887 (0.838–0.926)
*χ*^2^/*Z*	–	11.729	86.916	81.128	40.699	38.247	9.969
*P*	–	0.001	<0.001	<0.001	<0.001	<0.001	<0.001

ACR-TIRADS, American College of Radiology Thyroid Imaging Reporting and Data System; AUC, area under curve; NPV, negative predictive value; PPV, positive predictive value; SMI-TIRADS, ACR-TIRADS combined with SMI blood flow grades.

## Discussion

MTNs are benign TNs that show morphologic changes over time, which could result in misleading sonographic features suggestive of malignant nodules (23), leading in turn to potential over-management. In this study, most MTNs appeared as hypoechoic or very hypoechoic, solid nodules on conventional US imaging, similar to PTCs. The MTN lesions were <15 mm in diameter, and about 27% had ill-defined or irregular-lobulated/irregular margins on US imaging, possibly as a result of the collapse, stretching, or interruption of initially well-defined benign TNs (24). No MTNs showed extrathyroidal extension on US imaging in our study, and about 74% of MTNs had clear boundaries with a thyroid capsule, which helped to differentiate them from PTCs. Nearly half of MTNs included punctate echogenic foci, similar to microcalcifications inside PTCs, which comprise necrotic substances such as cholesterol crystallization and keratin (25). MTNs are challenging to discriminate from PTCs using conventional US; however, large comet-tail artifacts or macrocalcifications were more common in MTNs than in PTCs (30% vs 4%), while PTCs were more likely to have a taller-than-wide shape than MTNs (65% vs 44%), possibly due to the asymmetric fibrous healing associated with transverse shrinking (8). All of the above features helped to differentiate MTNs from malignancies in our study, consistent with the previous findings of Chen and coworkers (26).

**Figure 4:**
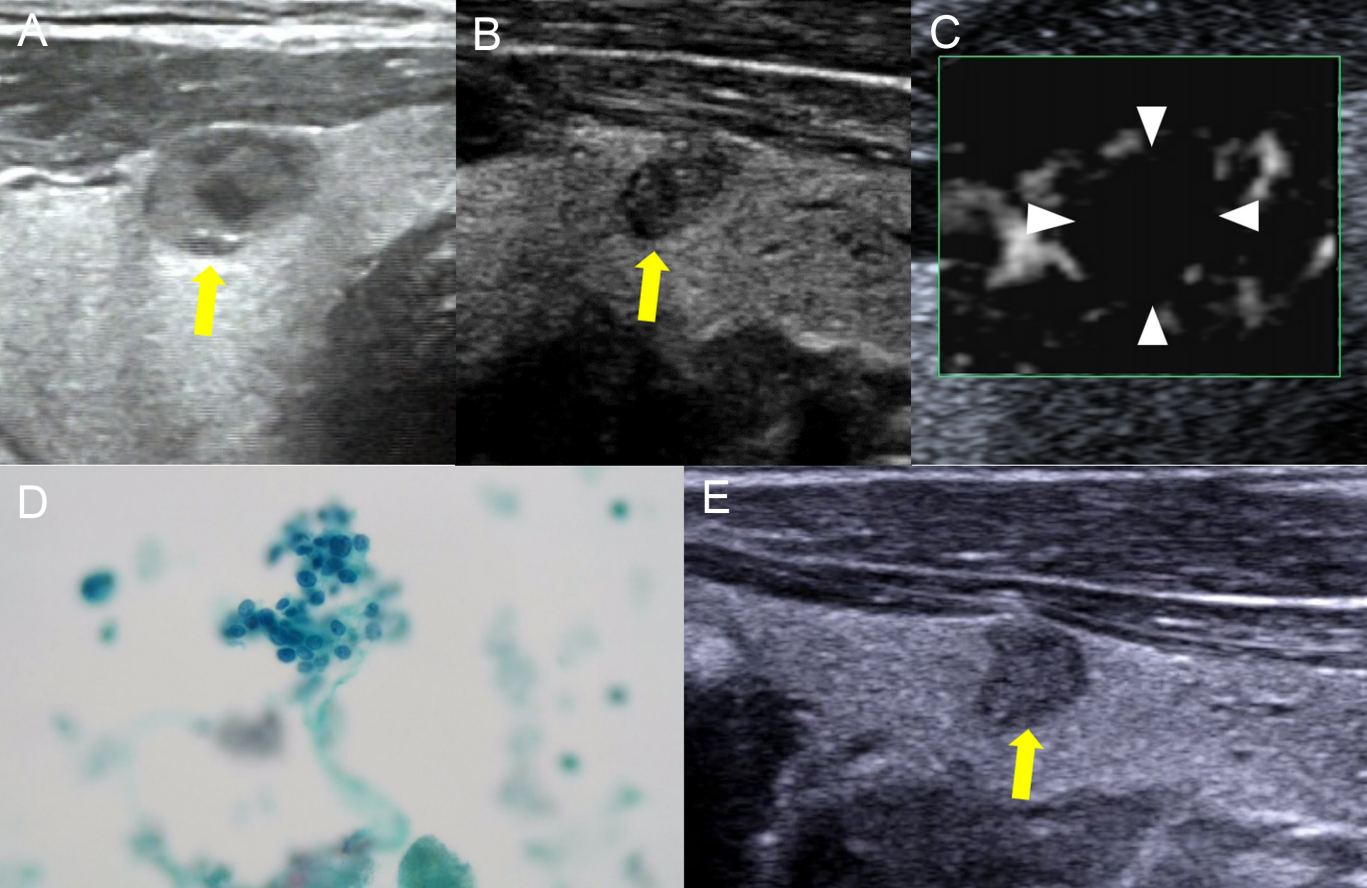
Images showed a solid TN in a 64-year-old man. The FNA diagnosis was a benign TN. (A) Conventional US imaging showed that there was a well-defined cystic and solid nodule in the thyroid gland (yellow arrow), and the maximum diameter of the nodule was 11 mm. (B) Two years later, the nodule became smaller, the maximum diameter of the nodule was 5 mm. There were three suspicious signs within the nodule (solis, irregular margin and microcalcification), and it was defined as ACR-TIRADS 5 (yellow arrow). (C) Monochrome superb microvascular imaging showed no blood flow signal inside the thyroid nodule, which was defined as grade 0. (D) Nuclear grooves and intranuclear inclusion bodies were not found in cell pathology, and thyroid follicular epithelial cells were easily to be seen. (E) The nodule was found to have no change in size and ACR-TIRADS category six months later.

**Figure 5:**
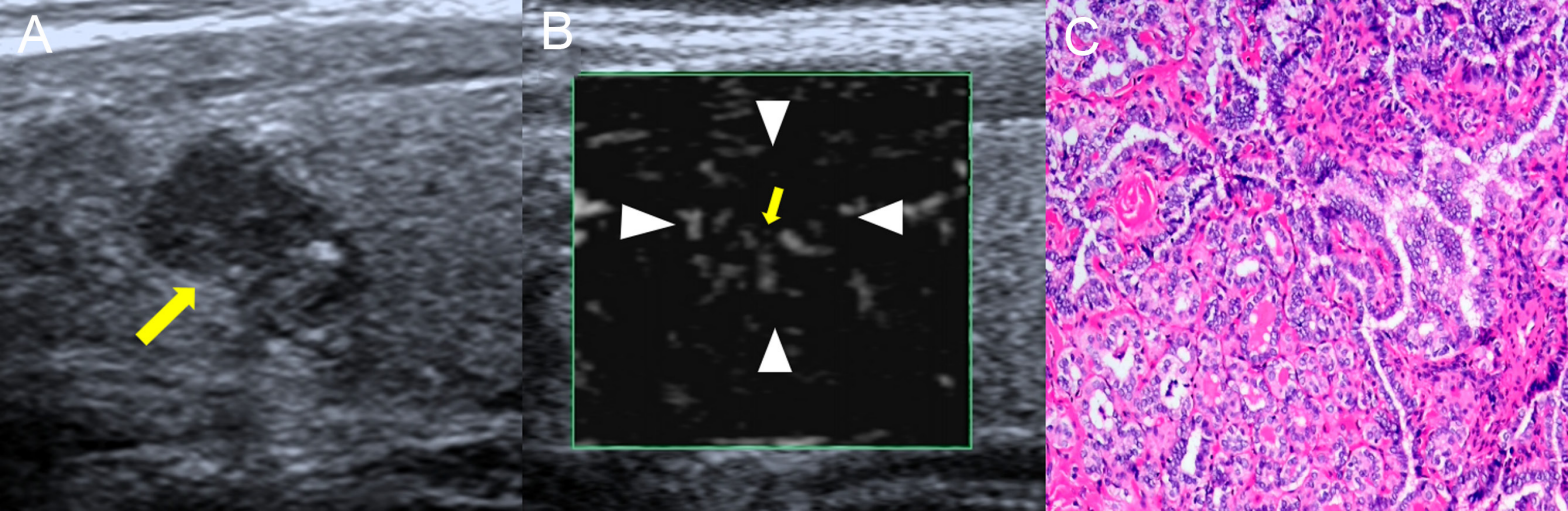
Images showed a solid TN in a 56-year-old female. The pathological diagnosis was a malignant TN. (A) Conventional US imaging showed that there was a ill-defined solid nodule in the thyroid gland (yellow arrow), and the maximum diameter of the nodule was 5 mm. (B) Monochrome superb microvascular imaging showed that there were four vessels within the nodule (yellow arrow), which was defined as grade 3. (C) The pathological result was papillary thyroid carcinoma.

In this study, however, it was difficult to distinguish MTNs from PTCs based on the ACR-TIRADS score using conventional US imaging (Fig. 4B and 5A), and 78.18% of MTNs had an ACR-TIRADS score of 5, which would result in unnecessary diagnostic surgery in patients with no clear clinical history, such as previous cystic TNs. Thus, there is a need for new techniques to improve the diagnostic accuracy of the ACR-TIRADS classification.

Cystic necrotic MTNs frequently exhibit a lack of vasculature, which can be explained by degeneration, shrinkage of cystic nodules with necrosis, and scant cells and tissues (27). In contrast, like most cancers, PTCs show increased and distorted vasculature, and the nodules thus have a rich blood supply. The blood-flow characteristics within the nodules can thus help to discriminate between MTNs and PTCs. It is difficult to differentiate the microvessels within TNs with CDFI, which is limited to distinguishing MTNs and PTCs by the internal blood flow feature of TNs (28). SMI is a novel Doppler technique that can overcome the shortcomings of color Doppler, and display the microcirculation with a higher resolution, and demonstrate improved microvascular blood and slow blood-flow signals within TNs, compared with CDFI (29). The fine perforating vessels inside the nodules can be used as an indicator for differentiating between benign and malignant TNs (21). The results of the current study showed that about 95% of MTNs had SMI blood flow grade 0–1 (Fig. 4C), and about 85% of PTCs had grade 2–3 (Fig. 5B), confirming that microvessels were less abundant in MTNs than in PTCs. Overall, 84.5% of MTNs were reclassified to TIRADS score 4 or 3, and 91.8% of PTCs were reclassified to TIRADS score 5 after combining SMI blood-flow grade. From the curve calculation, ACR-TIRADS showed poor diagnostic efficiency for distinguishing MTNs from PTCs (AUC 0.517) compared with SMI-TIRADS (AUC 0.887). The SMI-TIRADS mode not only yielded a higher AUC but also showed significantly higher sensitivity for differentiating MTNs from PTCs. These results indicate that SMI-TIRADS may help to reduce the misdiagnosis rate. We did not compare SMI and CEUS for microvascular imaging in this study, and further studies are needed to examine their efficiency and effectiveness.

## Limitations

This study has some limitations. The final diagnosis of MTN was used as the reference standard for cytologic examination, with at least 6 months of US follow-up; however, we did not conduct a final histopathologic examination to confirm benignity, and systematic close follow-up and FNA were not performed in all cases. In addition, a lower grade of SMI blood flow could not completely rule out the possibility of PTC because fibrous connective tissue hyperplasia in PTCs may prevent tumor neoangiogenesis and the detection of microvessels by SMI. Furthermore, this was a single-center study with a small sample size, and the data were thus inevitably biased. The high proportion of small TNs in this study may also have affected the results.

## Conclusion

The differential diagnosis of MTNs and PTCs based on US examination can be challenging because MTNs can display malignant features on US, resulting in a misleadingly high TIRADS score. SMI-TIRADS can provide more information for diagnosing nodules and can thus help to distinguish MTNs from PTCs. This, in turn, may help patients to avoid unnecessary surgical excision or FNA.

## Declaration of interest

The authors declare that they have no known competing financial interests or personal relationships that could have appeared to influence the work reported in this paper.

## Funding

This work was supported by the Zhuhai City Social Development Field Science and Technology Plan Project (number 2320004000146).

## Author contribution statement

JT: Data curation and analysis, methodology, validation, writing – original draft. JL: Investigation, methodology, validation, writing – review and editing. YL: Investigation, methodology. LW: Supervision. XC: Project administration, methodology, writing – review and editing. All authors read and approved the final draft of the manuscript.
